# A Polyphenol-Rich Extract of Olive Mill Wastewater Enhances Cancer Chemotherapy Effects, While Mitigating Cardiac Toxicity

**DOI:** 10.3389/fphar.2021.694762

**Published:** 2021-08-03

**Authors:** Adriana Albini, Marco M. G. Festa, Nadja Ring, Denisa Baci, Michael Rehman, Giovanna Finzi, Fausto Sessa, Serena Zacchigna, Antonino Bruno, Douglas M. Noonan

**Affiliations:** ^1^ Laboratory of Vascular Biology and Angiogenesis, IRCCS MultiMedica, Milan, Italy; ^2^ Cardiovascular Biology Laboratory, International Centre for Genetic Engineering and Biotechnology, Trieste, Italy; ^3^ Department of Pathology, ASST Settelaghi, Varese, Italy; ^4^ Department of Medicine and Surgery, University of Insubria, Varese, Italy; ^5^ Department of Medicine, Surgery and Health Science, University of Trieste, Trieste, Italy; ^6^ Laboratory of Innate Immunity, Unit of Molecular Pathology, Biochemistry and Immunology, IRCCS MultiMedica, Milan, Italy; ^7^ Immunology and General Pathology Laboratory, Department of Biotechnology and Life Sciences, University of Insubria, Varese, Italy; ^8^ Unit of Molecular Pathology, Immunology and Biochemistry, IRCCS MultiMedica, Milan, Italy

**Keywords:** polyphenols, cardioncology, cardio protection, cardio prevention, cardiotoxicity, heart, cancer

## Abstract

Cardiovascular toxicity remains one of the most adverse side effects in cancer patients receiving chemotherapy. Extra-virgin olive oil (EVOO) is rich in cancer preventive polyphenols endowed with anti-inflammatory, anti-oxidant activities which could exert protective effects on heart cells. One very interesting derivative of EVOO preparation is represented by purified extracts from olive mill waste waters (OMWW) rich in polyphenols. Here, we have investigated the anti-cancer activity of a OMWW preparation, named A009, when combined with chemotherapeutics, as well as its potential cardioprotective activities. Mice bearing prostate cancer (PCa) xenografts were treated with cisplatin, alone or in combination with A009. In an *in vivo* model, we found synergisms of A009 and cisplatin in reduction of prostate cancer tumor weight. Hearts of mice were analyzed, and the mitochondria were studied by transmission electron microscopy. The hearts of mice co-treated with A009 extracts along with cisplatin had reduced mitochondria damage compared to the those treated with chemotherapy alone, indicating a cardioprotective role. To confirm the *in vivo* results, tumor cell lines and rat cardiomyocytes were treated with cisplatin *in vitro*, with and without A009. Another frequently used chemotherapeutic agent 5-fluorouracil (5-FU), was also tested in this assay, observing a similar effect. In vitro, the combination of A009 with cisplatin or 5-FU was effective in decreasing prostate and colon cancer cell growth, while it did not further reduce growth of rat cardiomyocytes also treated with cisplatin or 5-FU. A009 cardioprotective effects towards side effects caused by 5-FU chemotherapy were further investigated, using cardiomyocytes freshly isolated from mice pups. A009 mitigated toxicity of 5-FU on primary cultures of mouse cardiomyocytes. Our study demonstrates that the polyphenol rich purified A009 extracts enhance the effect of chemotherapy *in vitro* and *in vivo,* but mitigates chemotherpy adverse effects on heart and on isolated cardiomyocytes. Olive mill waste water extracts could therefore represent a potential candidate for cardiovascular prevention in patients undergoing cancer chemotherapy.

## Introduction

Cancer therapy has made remarkable advances for the treatment of solid and hematological tumors, leading to significant progresses in the reduction of tumor recurrences (Albini et al., 2010; Albini et al., 2012a; Angsutararux et al., 2015; Conway et al., 2015; Focaccetti et al., 2015; Curigliano et al., 2016; Polonsky and DeCara, 2019). Although the introduction of different antineoplastic agents in the clinic, such as monoclonal antibodies and tyrosine kinase inhibitors, has significantly augmented life expectancy (Senkus and Jassem, 2011), cardiovascular toxicity remains a major clinical concern, sometimes generating higher morbidity and mortality than tumor recurrences (Senkus and Jassem, 2011). Cardiovascular toxicities, defined as “toxicities affecting the heart”, are among the most frequent undesirable effects of cancer chemotherapy. Major effects of chemotherapy-induced cardiovascular toxicities include arrythmias, myocardial ischemia, coronary artery diseases, hypertension, and myocardial dysfunctions (Polonsky and DeCara, 2019).

A major problem in the manifestation of clinically evident cardiotoxic events is the fact that they are often asymptomatic, and therefore negatively impact the cardiological prognosis of cancer patients, as well as significantly limits applicable treatment options (Albini et al., 2010 ; Albini et al., 2012a ; Angsutararux et al., 2015 ; Conway et al., 2015; Focaccetti et al., 2015; Curigliano et al., 2016; Polonsky and DeCara, 2019). In fact, even minor cardiac dysfunctions significantly restrict the choice of therapeutic programs, forcing the selection of those considered less aggressive and, as such, potentially less effective (Albini et al., 2010; Albini et al., 2012a; Angsutararux et al., 2015; Conway et al., 2015; Focaccetti et al., 2015; Curigliano et al., 2016; Polonsky and DeCara, 2019). Occurrence of chemotherapy-induced cardiotoxicity is continuously increasing, as a consequence of the growing number of patients undergoing chemotherapy and the introduction of new, more aggressive, anticancer drugs, often administered in combination with other toxic compounds (Albini et al., 2010; Albini et al., 2012a; Angsutararux et al., 2015; Conway et al., 2015; Focaccetti et al., 2015; Curigliano et al., 2016; Polonsky and DeCara, 2019).

This knowledge suggested that a strict dialogue between the oncologists and the cardiologists is necessary, when selecting the proper chemotherapy intervention, as well as cardiac monitoring in cancer patients, bringing to a new discipline termed cardio-oncology (Albini et al., 2010).

Mitochondria represent the metabolic engine, governing and sensing the cellular energy requirements during physiological and pathological conditions (Vringer and Tait, 2019; Missiroli et al., 2020). Cardiomyocytes strongly depend on mitochondria for energy requirements. The maintenance of mitochondrial membrane potential is crucial to supply gradients for ATP synthesis (Bhatti et al., 2017). Oxidative stress represents a major hallmark of age- and chronic inflammatory-related disorders and significantly impacts on mitochondrial functionality (Bhatti et al., 2017). Generation of reactive oxygen species (ROS) (Toric et al., 2019; Fiorentino et al., 2020; Li et al., 2020; Cocetta et al., 2021; Oruganti and Meriga, 2021; To and Cho, 2021) and mitochondrial damage are major drivers of chemotherapy-induced cardiotoxicities (Hahn et al., 2014; Ichikawa et al., 2014; Nitiss and Nitiss, 2014; Zhang et al., 2020).

Polyphenols can acts as anti-cancer agent when combined with chemotherapy (Albini et al., 2007; Albini et al., 2012b; Rossi et al., 2014; Bassani et al., 2016; Albini et al., 2019; Toric et al., 2019; Fiorentino et al., 2020; Li et al., 2020; Cocetta et al., 2021; Oruganti and Meriga, 2021; Sartori et al., 2021) and can exhibit cardio protective effects (Cheng et al., 2017; Zheng et al., 2018; Choy et al., 2019; Martínez-González et al., 2019; Poti et al., 2019; Atale et al., 2020). Polyphenols can overcome multidrug resistance (El-Readi et al., 2021). Chemotherapy with Doxorubicin associates with cardiovascular toxicities; polyphenols such as resveratrol and curcumin can abate this toxicity (Rezk et al., 2006; Shakibaei et al., 2014; Kalyanaraman, 2020). Polyphenols act as anti-oxidants, by contrasting the generation of ROS that drive cellular and mitochondrial damage.

It has been widely demonstrated that adherence to the Mediterranean diet is associated with reduced risk of developing cardiovascular diseases. In recent decades, numerous epidemiological and interventional studies have confirmed this observation, underlining the close relationship between the Mediterranean diet and cardiovascular diseases (Grosso et al., 2014; Billingsley and Carbone, 2018; Estruch et al., 2018). In this context, extra-virgin olive oil (EVOO), the most representative component of this diet, seems to be important in reducing the incidence of cardiovascular events, including myocardial infarction and stroke (Nocella et al., 2018). Current research on the beneficial effect of EVOO is focused on defining its protective effects against cardiovascular risk factors, such as inflammation, oxidative stress, coagulation, platelet aggregation, fibrinolysis, and endothelial or lipid dysfunction. A further approach is based on the modulation of conditions that predispose people to cardiovascular events, such as obesity, metabolic syndrome or type 2 diabetes mellitus, and chemotherapy (Estruch et al., 2018; Marcelino et al., 2019; Martínez-González et al., 2019; Mazzocchi et al., 2019; Nediani et al., 2019). The protective activity of EVOO results from the high levels of phenolic compounds, monounsaturated fatty acids (MUFA) and other minor compounds present in EVOO (Nocella et al., 2018).

Industrial EVOO processing is associated with the generation of large volume of liquid waste products, termed olive mill wastewater (OMWW) (El-Abbassi et al., 2012; Vougogiannopoulou et al., 2015). OMWW are rich in water soluble polyphenols, endowed with anti-bacterial, anti-antioxidant, cytoprotective activities, (Schaffer et al., 2010; Abu-Lafi et al., 2017; Belaqziz et al., 2017), thus representing a valid waste product to be repositioned in the market (Taticchi et al., 2019a; Taticchi et al., 2019b).

Here, we investigate the potential cardioprotective activities of a polyphenol-rich, EVOO-derived polyphenol extracts (A009), derived from olive mill wastewater (OMWW). A009-extracts have been reported to exhibit chemopreventive and angiopreventive properties, *in vitro* and *in vivo*, in different cancer types (Baci et al., 2019; Gallazzi et al., 2020).

We examined A009 effects on tumor growth, when combined with a chemotherapeutic agent and evaluated the effects of the combination on the heart and cardiomyocytes, at both cellular and molecular level, using *in vivo* (mice bearing prostate tumors) and *in vitro* models.

## Materials and Methods

### Chemicals

Cis-Diammine platinum dichloride (Cis-Pt) and 5-Fluorouracil (5FU), all purchased by SIGMA Aldrich were dissolved in dimethyl sulfoxide (DMSO) and used for *in vitro* experiments as detailed below. 3-(4,5-dimethylthiazol-2-yl)-2,5-diphenyltetrazolium bromide (MTT) was purchased by SIGMA Aldrich and resuspended at 5 mg/ml. A009 polyphenol -rich extract, derived from olive mill wastewater (OMWW) processing, were provided by Azienda Agricola fattoria La Vialla, Castiglion Fibocchi, Arezzo Italy.

### Preparation of A009 Extracts

The A009 was obtained from the OMWW derived from the processing of EVOO. Extraction procedures and polyphenol quantification has been previously published (Bassani et al., 2016, Baci et al., 2019). The polyphenol composition is not altered, following different years of cultivars (Bassani et al., 2016; Baci et al., 2019). Polyphenol content of the A009 extract is showed in Supplemental Table S1, Supplementary Figure S1 and has been published (Bassani et al., 2016, Baci et al., 2019).

### Cell Line Culture and Maintenances

The human prostate cancer (PCa) cell lines DU-145, 22Rv1 and the colorectal cancer cell line HT29 (all purchased by ATCC) were maintained in RPMI 1640 medium, supplemented with 10% Fetal Bovine Serum (FBS) (Euroclone), 2 mM l-glutamine (Euroclone), 100 U/ml penicillin and 100 μg/ml streptomycin (Euroclone), at 37°C, 5% CO_2_. The rat cardiomyocyte cell line H9C2 (PromoCell) was maintained in Myocyte Growth Medium plus Myocyte supplements mix (PromoCell), addition with 10% Fetal Bovine Serum (FBS) (Euroclone), 2 mM l-glutamine (Euroclone), 100 U/ml penicillin and 100 μg/ml streptomycin (Euroclone), at 37°C, 5% CO_2_. Cells were routinely screened for eventual *mycoplasma* contaminations.

### Detection of Cardioprotective Activities *in Vivo* Tumor Xenograft Models

We used a mouse model of prostate cancer to determine whether co-treatment with the chemotherapeutic agent cisplatin and A009 extract could exert a protective effect on the hearts of the treated animals. The effects of the A009 extracts in inhibiting prostate cancer (PCa) tumor cell growth was assessed using an *in vivo* xenograft model. 5-week-old male Nu/MRI nude mice (from Charles River) were used, with four animals per experimental group. Animals were housed in a conventional animal facility with 12:12 h light dark cycles and fed ad libitum. Animals were subcutaneously injected into the right flank with 2.5 × 10^6^ 22Rv1 cells or DU-145 cells, in a total volume of 300 μL, containing 50% serum free RMPI 1650, and 50% 10 mg/ml reduced growth factor Matrigel (Corning) with or without A009 (dilution 1:250). From day 0 animals received A009 daily (dilution 1:250), in the drinking water. When tumors were palpable, mice received Cisplatin, 7 mg/kg *i. p*, twice a week. At day 27, the tumor cell growth was stopped, tumors were excised, weighted and tumor volume was measured with a caliper and determined using the formula (W^2^ × L)/2. Hearts were surgically removed from animals and used for transmission electron microscopy analyses.

All the procedures involving the animals and their care were performed according to the institutional guidelines, in compliance with national and international law and guidelines for the use of animals in biomedical research and housed in pathogen-free conditions. All the procedures applied were approved by the local animal experimentation ethics committee (ID# #06_16 Noonan) of the University of Insubria and by the Italian Health Ministry (ID#225/2017-PR).

### Transmission Electron Microscopy Analysis of Murine Hearts

Hearts were surgically excised from sacrified animals and extensively washed in PBS. Heart sections were obtained using a scalpel and then placed in fixing solution for TEM processing (2% PFA, 2% glutaraldehyde), finally post-fixed using 1% osmium tetroxide and embedded in an Epon-Araldite resin. Following exposure to uranyl acetate and lead citrate, thin sections were analyzed by TEM, using a Morgagni electron microscope (Philips) at 3500X magnification, to detect mitochondrial alterations in terms of morphology, size, organization, and quantity. The number of altered mitochondria per section, exhibiting altered morphology/shape, was counted using the ImageJ software.

### Combination Effect of Chemotherapy and A009 on Cancer Cell Lines

To investigate whether the A009 extract could synergize with chemotherapy, the prostate cancer DU-145 cell line or the colorectal cancer HT-29 cell line were treated with Cis-Pt 100 µM or 5-FU 100 μM, respectively, alone or in combination with A009 L3 or L4 extracts, for 24–72 h. Detection of cell viability was determined by MTT (3-[4,5-dimethylthiazole-2-yl]-2,5-diphenyltetrazolium bromide) assay, on 3,000 cardiomyocytes/well, seeded into a 96 well plate.

### Effects of A009 Extracts on Adult Rat Cardiomyocyte

To evaluate the effects of the A009 extracts on chemotherapy induced cardiotoxicity, after preliminary experiment to assess dosages, adult rat cardiomyocyte H9C2 cells were treated with 5-FU 100 µM or Cis-Pt 100 μM, alone or in combination with A009 L3 or L4 extracts, for 24–72 h. The schedule treatments included a prevention approach by pre-treating cardiomyocyte with A009 L3 and L4 extracts at T24 to T48 h, subsequently A009 L3 or L4 extracts were removed, and wells were auditioned with fresh medium containing Cis-Pt 100 µM or 5-Fu 100 µM. Detection of cell viability was determined by MTT assay descripted in 2.6.

### Isolation of Neonatal Murine Cardiomyocytes

Cardiomyocytes were isolated from neonatal C57/Bl6 mice at 2 days after birth as previously described, with minor modifications (Zacchigna et al., 2018). Briefly, hearts were removed and cleaned in calcium and bicarbonate-free Hanks’ balanced salt solution with HEPES (CBFHH, containing 137 mM NaCl, 5.36 mM KCl, 0.81 mM MgSO_4_ 7H_2_O, 5.55 mM dextrose, 0.44 mM KH_2_PO_4_, 0.34 mM Na_2_HPO_4_ 7H_2_O, and 20.06 mM HEPES). Excess blood and valves were removed, and hearts were diced. The tissue was then enzymatically digested using CBFHH supplemented with 1.75 mg/ml of Trypsin (BD Biosciences) and 20 mg/ml of DNAse I (Sigma). Tissue was digested for 3 h, with cells harvested into fetal bovine serum (FBS) every 10 min to stop the digestion. Cells were then filtered using a 40 μm cell strainer and pre-plated for 2 h to remove contaminating fibroblasts. Finally, cardiomyocytes were collected and seeded on tissue culture plates treated for primary cultures. Cells were cultured in Dulbecco’s modified Eagle medium 4.5 g/L glucose (DMEM, Life Technologies) supplemented with 5% FBS, 20 mg/ml vitamin B12 (Sigma), 100 U/ml penicillin and 100 mg/ml streptomycin (Sigma).

### Effects of A009 Extracts on Neonatal Murine-Derived Cardiomyocytes

To evaluate the effect of the A009 extract on cardiomyocyte viability *in vitro*, 30,000 cardiomyocytes/well were seeded into a 96 well plate. One day after plating, cells were treated with L3 and L4 A009 extracts, dilution of 1:800, for 24 h. On day 2, cells were treated with 4.6 µM of 5-Fluorouracil. Following 24 and 48 h, cells were fixed and stained using anti-Cardiac Troponin I antibody (Abcam, ab47003, dilution of 1:200) and Hoechst 33,342 (Invitrogen, H3570, dilution of 1:5000). The number of cardiomyocytes for each time point was counted in three independent experiments.

## Results

### Cardioprotective Activities of A009 Extracts *in Vivo* Models of Cardiotoxicity Induced by Anticancer Drug

We used a mouse model of prostate cancer xenographt to determine the A009-extract effect on tumors and the hearts of mice treated with the chemotherapeutic agent cisplatin. During the treatment schedule, we did not observe behavioral changes, alterations in food intake, water consumption, or dejections by the animals included in all the experimental groups of the study (Table 1).

**TABLE 1:** Monitoring of healthy conditions during *in vivo* treatments.

	NT	Cisplatin (7 mg/kg)	A009 1:250	Cisplatin (7 mg/kg) + A009 1:250
Skin peeling	0 (10)	5 (9)	1 (10)	2 (10)
Dehydration	0 (10)	0 (9)	0 (10)	0 (10)
Alterations in water consumption	0 (10)	0 (9)	0 (10)	0 (10)
Alterations in food consumption	0 (10)	0 (9)	0 (10)	0 (10)
Urine	0 (10)	0 (9)	0 (10)	0 (10)
Feces	0 (10)	0 (9)	0 (10)	0 (10)

The healthy state on mice receiving single agent (A009, dilution 1:250) alone, or Cisplatin (7 mg/kg) alone, or the combinations of Cisplatin with the A009 extract was daily monitored. As readout of clinical parameters, the presence of skin peeling, dehydration, alterations of water and food consumption, alteration in solid (feces) and liquid (urine) dejections are showed. Data are presented as (number of events)/total animal per experimental conditions.

**FIGURE 1:**
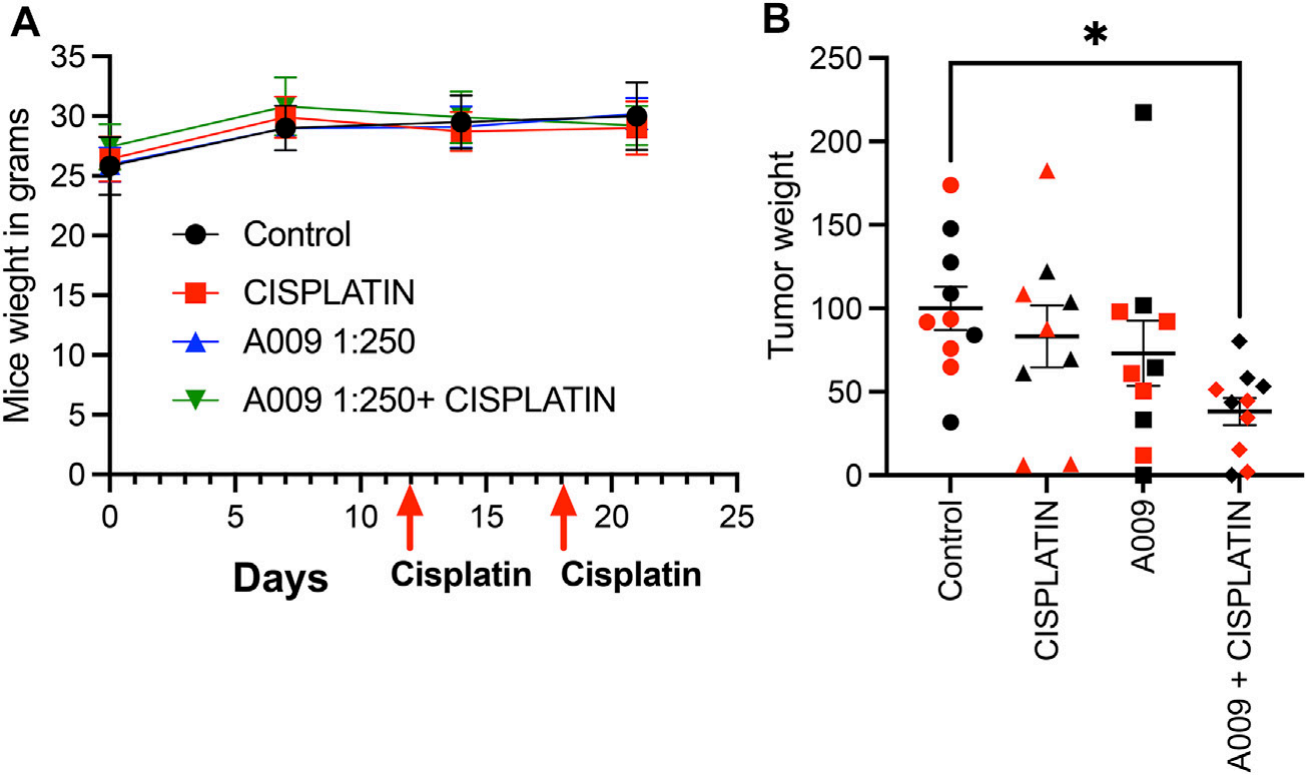
Dietary administration of the A009 extract, in combination with chemotherapy, resulted in both synergism by reducing tumor weight. Dietary administration (drinking water) of A009 extracts synergizes with chemotherapy by reducing tumor weight *in vivo*. In panel **(A)** the red arrows indicate the day of administration dose of cisplatin (7 mg/kg), the mice weights did not change. In panel **(B)**, the effects of the combination of A009 extract with cisplatin (7 mg/kg), was determined by measuring the weight of the tumors excised from the orthotopic *in vivo* model of prostate cancer cells DU-145 (red), 22Rv1 (black), normalized to the control group tumor weight. Data are showed as mean ± SEM, one-way ANOVA, *p < 0.05.

Animals receiving the different treatment did not show weight loss during the tumor cell growth kinetic (Figure 1A). Interesting, A009 also reduced the skin peeling induced by cisplatin treatments (from 5/9 mice to (2/10 mice) (Table 1). We found that the combination of cisplatin with the A009 extract synergized by further reducing the PCa cell tumor weight, as compared to the treatment with cisplatin alone (Figure 1B). The macroscopical/morphological inspection did not reveal detectable differences amongst the hearts of the various experimental groups (data not shown).

**FIGURE 2:**
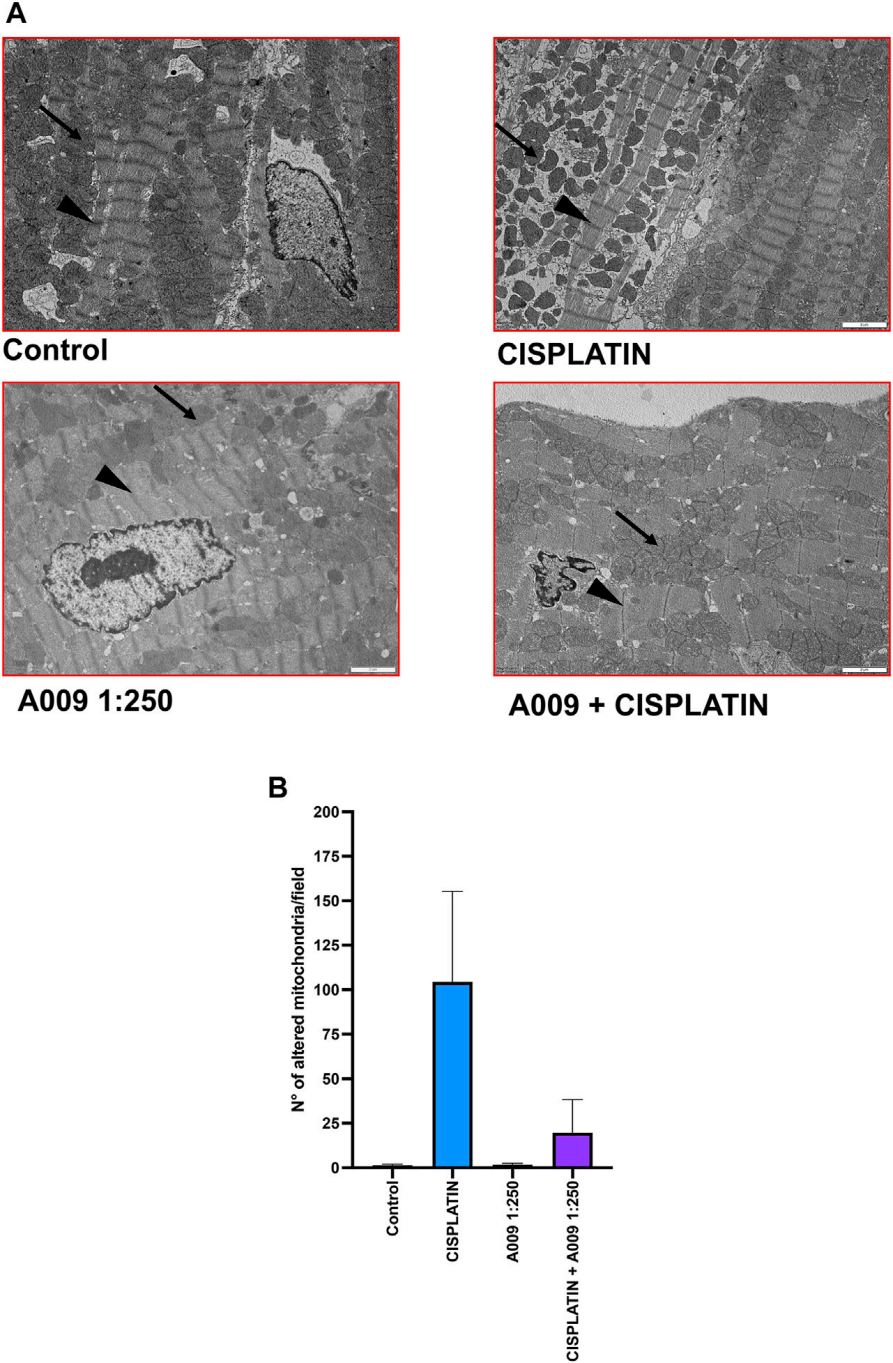
A009 cardioprotective activities against cisplatin-induce cardiotoxicity *in vivo*. Mitochondria number, shape/morphology and color was monitored, by transmission electron microscopy (TEM) on hearts from mice treated with cisplatin alone (7 mg/kg), A009 extract (dilution 1:250, in drinking water) or the cisplatin-A009 extract combination. **(A)** representative TEM micrographs (arrows for the mitochondria, arrowheads for the z-line). **(B)** graph bars showing the count of altered mitochondria per experimental condition. Data are showed as mean ± SEM.

However ultrastructural analysis, using transmission electron microscopy (TEM), showed that animals treated with cisplatin which received also the A009 extract have a reduced number of damaged mitochondria (showing a rounder shape and having mitochondrial cristae better organized and higher in number), as compared to the hearts of mice treated with cisplatin only (Figures 2A,B). We also observed a more regular muscle myosin and actin fiber disposition in the hearts of animals treated with A009 and cisplatin as compared to those treated with cisplatin alone. Henatoxylin/Eosin analysis, by optical microscopy, showed no alterations of cardiomyocytes; inflammation and fibrosis were observed in hearts treated with cisplatin, nor in those treated with A009 with and without cisplatin. Therefore, it is confirmed that in these cases the electron microscopy data are the only ones able to demonstrate cellular suffering (in particular those relating to mitochondria).

### A009 Activities Against Tumor Cell Lines and Heart Cell Lines

**FIGURE 3:**
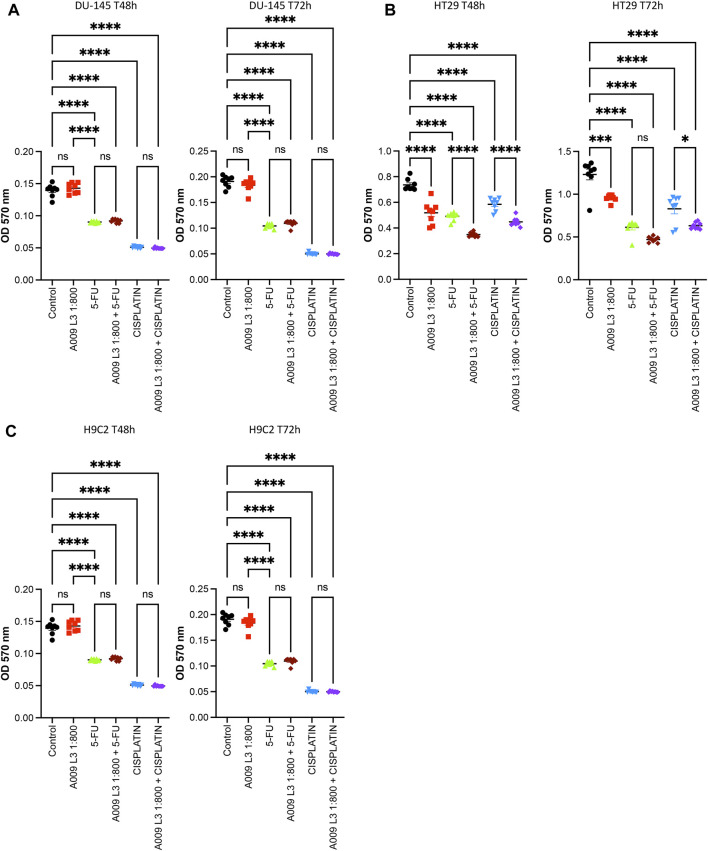
Activities of A009 extracts combined with chemotherapy on tumor cells and cardiomyocytes. A009 (batch L3) deceases the proliferation rate of tumors cells *in vitro* [**(A)**: DU-145 PCa and **(B)**: HT29 CRC and has additive effects on the cisplatin and the 5-Fluorouracil (5FU) effects. The cardiomyocytes proliferation rate is not affected by A009 alone **(C)**, and reduced proliferation by 5FU and cisplatin is not further decreased by A009 *in vitro*. Control: vehicle control. Data are showed as mean ± SEM, one-way ANOVA, *p < 0.05, **p < 0.01, ***p < 0.001, ****p < 0.0001.

Cisplatin and 5-FU treatment in vitro decreased both prostate (Figure 3A) and colon cancer (Figure 3B) cell growth. The proliferation of the tumor cells treated with A009 was also significantly different from the control vehicle. A009 enhanced the effect of the cisplatin and 5-FU alone (Figures 3A,B, Supplementary Figure S2) on prostate and colon cancer cells. 5-FU and cisplatin were toxic for rat cardiomyocytes, while the A009 was not. Furthermore, A009 in combination with Cisplatin or 5FU did not enhance the growth reduction (Figure 3C) induced by the chemotherapics. Therefore, while A009 significantly decreased tumor cell proliferation and exhibited additive effect with cisplatin and 5-FU, it did not affect cardiomyocyte growth and it did not enhance toxicity of cisplatin and 5-FU (Figure 3C).

### Protective Activities of A009 Extracts on Neonatal Murine Cardiomyocytes

**FIGURE 4:**
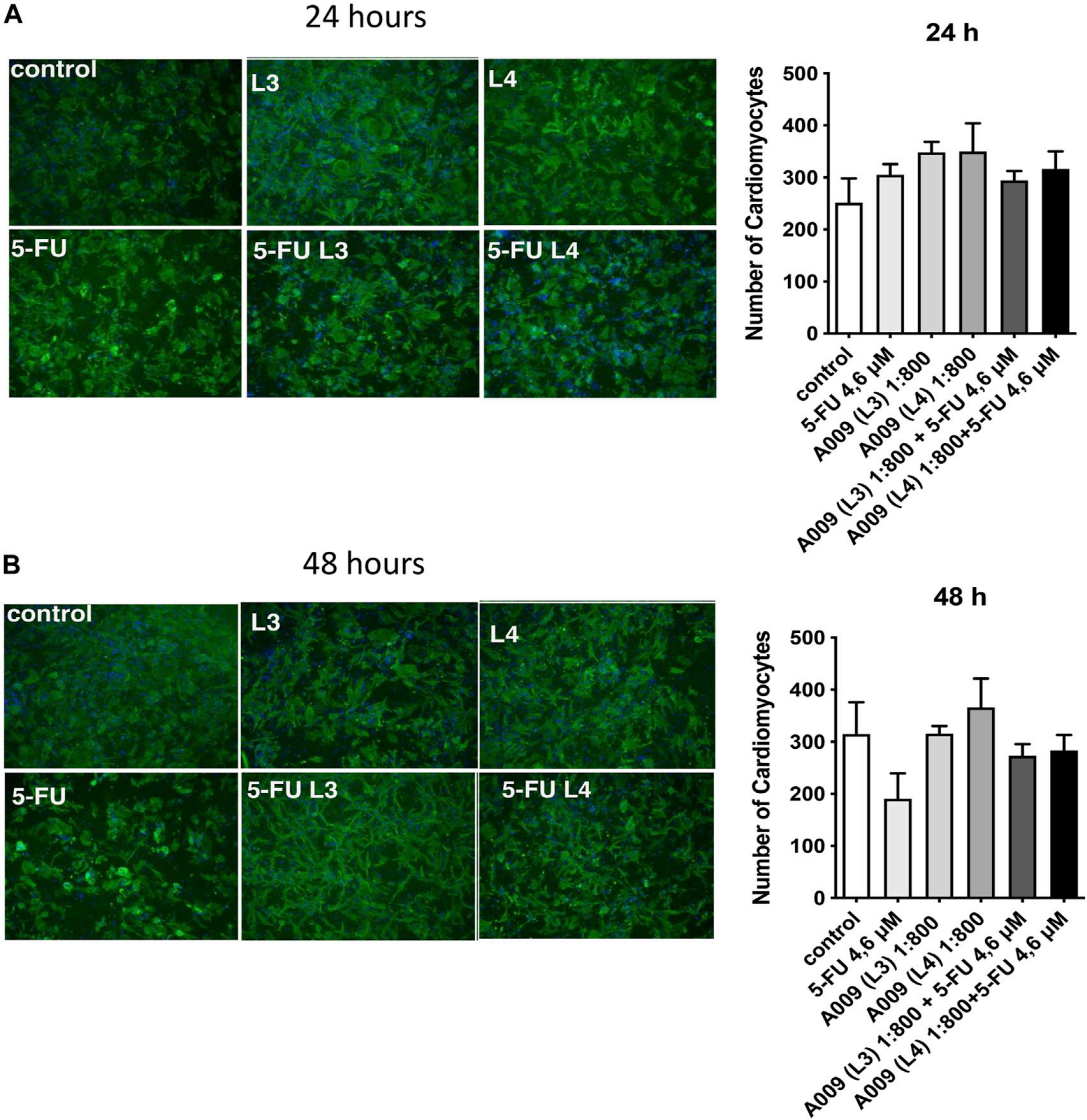
Protective activities of A009 extracts on neonatal murine cardiomyocytes. The cardioprotective effects of the A009 extract on chemotherapy induced cardiotoxicities was assessed, *in vitro*, on neonatal murine cardiomyocytes. Neonatal murine cardiomyocytes were exposed for 24 h **(A)** and 48 h **(B)** to 5-FU (4.6 μM) alone, A009 extracts (dilution 1:800, batches L3 or L4) alone, or the combination of the 5-FU and A009 extracts (dilution 1:800, batches L3 or L4). Number of cardiomyocytes was not affected by 5FU after 24 h treatments, while the OMWW extract improved their number to a slight but significative percent. After 48 h 5FU strongly decreased cardiomyocyte number to 60%. A009 alone did not lower the quantity of cardiomyocytes, and even slightly increased it as in the 24 h (in the L4 formulation). Addition to L3 and L4 together with the cytotoxic drug counteracted 5FU effect at 48 h. Data are showed as mean ± SEM. 5-FU: 5-fluoro-Uracile; L3/L4: A009 batch extract; Control: vehicle control; 5-fluorouracil.

We observed a cardioprotective effect of the A009 extracts on neonatal murine cardiomyocytes, following co-treatment with the chemotherapeutic drug 5-FU (Figure 4). The protective effect of the A009 extracts was studied by determining the number of viable cardiomyocytes, following 24 h (Figure 2A) and 48 h (Figure 2B) of treatment. At the early time point of 24 h, A009 showed a cardioprotective effect in basal conditions, and was slightly protective against 5-FU (Figure 4A). After 48 h, A009 was consistently cardioprotective also against 5-FU (Figure 4B).

## Discussion

Cardiovascular toxicities still remain a major challenge in clinical oncology (Albini et al., 2010; Senkus and Jassem, 2011; Albini et al., 2012a; Angsutararux et al., 2015; Conway et al., 2015). While chemotherapeutic agents efficiently target malignantly transformed cells, they simultaneously induce cell death of healthy cells (Albini et al., 2010; Senkus and Jassem, 2011; Albini et al., 2012a; Angsutararux et al., 2015; Conway et al., 2015). The cardiovascular system is the major off target of anti-neoplastic drugs (Albini et al., 2010; Senkus and Jassem, 2011; Albini et al., 2012a; Angsutararux et al., 2015; Conway et al., 2015). The patients treated with 5-FU can develop angina, acute myocardial infarction, Takotsubo and Raynaud’s syndrome as adverse effects, while cisplatin receiving patients can show angina, acute myocardial infarction, hypertension, Raynaud’s syndrome, Raynaud’s Stroke or peripheral arterial disease (Herrmann et al., 2016) as side effects. Most of the studies on chemotherapy agents were performed *in vitro* in cardiomyocytes and *in vivo* on the heart (Ma et al., 2020; Kim and Choi, 2021).

Mimicking a scenario closer to the clinic, we tested the cardioprotective properties of the A009 extract in an *in vivo* murine model of prostate tumor xenograft treated with cisplatin, a chemotherapy agent associated with cardiotoxicity and mitotoxicity (Varga et al., 2015; Ma et al., 2020). C57/Bl6 tumor bearing mice mice treated with cisplatin for 1 week developed myocardial contractile dysfunction; transmission electron microscopy revealed ultrastructural abnormalities of the mitochondria (Ma et al., 2010; Varga et al., 2015). Mice subcutaneously injected with the DU-145 prostate cancer cell line, co-treated with A009 and the chemotherapeutic drug cisplatin, showed a reduced number of abnormal and damaged mitochondria, as compared to those treated with cisplatin alone. Mitochondria have an essential role in myocardial tissue homeostasis (Varga et al., 2015; Brown et al., 2017) and diverse chemical compounds and chemotherapy drugs have been known to directly or indirectly modulate cardiac mitochondrial function (Gogvadze et al., 2009; Gorini et al., 2018). Mitochondrial oxidative stress and dysfunctions are common mechanisms in cardiotoxic effects (Altena et al., 2009; El-Awady et al., 2011; Ichikawa et al., 2014; Nitiss and Nitiss, 2014; Dugbartey et al., 2016; Brown et al., 2017). Cisplatin was tested *in vitro* on DU145 prostate cancer cell lines, as well as on HT29 colonc cancer cells, alone or in combination with A009 and its effects compared to those on rat cardiomyocytes.

Most of the cytotoxic activities of chemotherapeutic agents on normal cells are due to the induction of exacerbated oxidative stress, through the generation of both ROS and reactive nitrogen species (RNS) (Angsutararux et al., 2015; Zhang et al., 2018). Agents such as anti-inflammatory, anti-oxidants, able to counteract these effects, can be used to reduce side effects by chemotherapeutics and can be easily tolerated by oncologic patients and administered by dietary regimen (Kaiserová et al., 2007; Vincent et al., 2013). Many dietary polyphenols demonstrate anti-oxidant and cytoprotective properties (Krajka-Kuźniak et al., 2009; Polk et al., 2013; Baranowska and Bartoszek, 2016; Sara et al., 2018). We tested the ability of a polyphenol-rich purified extract of OMWW, termed A009, to protect from cardiovascular damages induced by the anti-cancer agent cis-platin, in vivo and in vitro.

5-FU is also a common cancer chemotherapeutic agent. The 5-FU cytotoxic action on cardiomyocytes results in mitochondrial dysfunctions (Eskandari et al., 2015). ROS scavengers, anti-oxidants can prevent, mitochondrial permeability induced 5FU in this study (Eskandari et al., 2015). In a previous study, we demonstrated that human cardiomyocytes exposed to 5-FU *in vitro* acquire a senescent phenotype and undergo autophagy (Focaccetti et al., 2015). While A009 significantly decreased tumor cell proliferation and had additive effect with cisplatin and 5-FU, it did not affect cardiomyocyte growth as single treatment and did not enhance toxicity of cisplatin and 5-FU. Based on these results, we investigated the effects of the A009 extracts also on fresh cardiomyocytes isolated from neonatal mice. In these experiments, we validated 5-FU cardiotoxic activities (Albini et al., 2010; Polk et al., 2013; Angsutararux et al., 2015; Focaccetti et al., 2015; Sara et al., 2018). We observed that cardiomyocytes co-treated with the A009 extracts and the chemotherapeutic drug 5-FU exhibited less reduction of the number of cardiomyocytes, as compared with the drug alone. This rescue was maintained from 24 to 48 h of cardiomyocyte culture and treatment and potentially related to the antioxidant polyphenols present in the A009 extracts.

## Conclusion

Here, we demonstrated that the A009 extracts, although additive in cancer therapy, do not have cardiotoxic effects, and can actually mitigate chemotherapy-induced cardiotoxicity. One of the effects, detected by transmission electron microscopy on hearts of treated mice, suggests mitochondrial protection and anti-oxidant capabilities of A009.

Our study demonstrates that a polyphenol rich purified OMWW extract can be placed as valid candidate for combination with chemotherapy (additive effects) while protecting the heart from chemotherapy-associated cardiovascular toxicities.

## Data Availability

The raw data supporting the conclusions of this article will be made available by the authors, without undue reservation.
