# Supplementary material for: A Polyphenol-Rich Extract of Olive Mill Wastewater Enhances Cancer Chemotherapy Effects, While Mitigating Cardiac Toxicity
Source: Front Pharmacol. 2021 Aug 3;12:694762. doi: 10.3389/fphar.2021.694762 (PMC8381749; doi:10.3389/fphar.2021.694762)
Supplement: Supplementary file 1 [file DataSheet1.DOCX]

Supplementary Material

# Supplementary Figure 1

| PHENOLIC COMPOUND | A009 L3 (g/L) | A009 L4 (g/L) |
| --- | --- | --- |
| Hydroxytyrosol | 5.72 | 5.50 |
| Hydroxytyrosol glucoside | 1.69 | 1.91 |
| Verbascoside | 1.32 | 1.07 |
| 6’-p-coumaroyl secologanoside | 0.40 | 0.35 |
| b-hydroxyverbascoside isomer 1 | 0.14 | 0.23 |
| b-hydroxyverbascoside isomer 2 | 0.17 | 0.23 |
| Chlorogenic acid | 0.10 | 0.13 |
| Caffeoyl ester of secologanoside | 0.20 | 0.23 |
| Decarboxymethyloleuropein aglycon | 0.28 | 0.16 |
| Oleouropein aglycon | 0.22 | 0.21 |

**L4**

**L3**

**Supplementary Figure 1: Phenolic composition of A009 was obtained by HPLC-DADMS-MS.** Samples were analyzed by HPLC with UV–vis and MS detection. The identification of phenolic compounds from samples was carried out as previously reported by interpreting their mass spectra determined via LC-MS-MS and comparing to data reported in literature identified the compounds**.**

## Supplementary Figures 2

**L4**


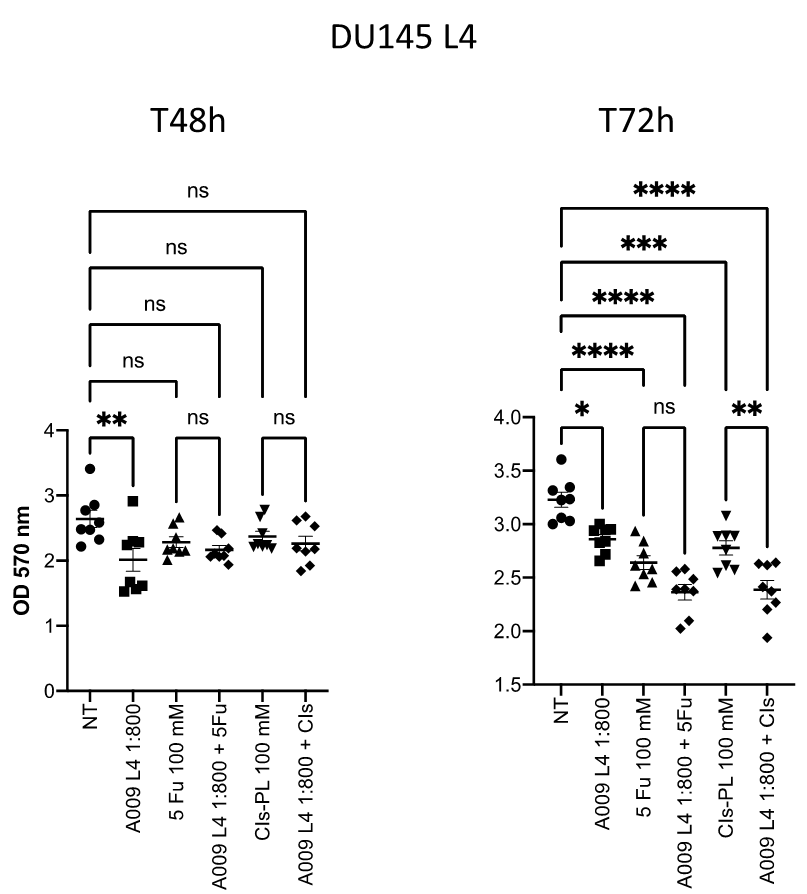


**Supplementary Figure 2: Activities of a second A009 batch on DU-145 PCa tumor cell line.** A009 (batch L4) decrease the proliferation rate of DU-145 PCa tumors line cells *in vitro* and has additive significantly effects on the cisplatin and trend towards 5-FU. Data are showed as mean ± SEM, one-way ANOVA, **p<0.01, ***p<0.001, ****p<0.0001.
